# AI-Driven Lifestyle Interventions for Reducing Diabetes Risk: Systematic Review

**DOI:** 10.2196/104940

**Published:** 2026-10-15

**Authors:** Obinna I Ekwunife, Tyler Nauta, Raphael A Fraser, Jennifer A Campbell, Gloria Aidoo-Frimpong, Rebekah J Walker, Leonard E Egede

**Affiliations:** 1 Division of Population Health, Department of Medicine Jacobs School of Medicine and Biomedical Sciences University at Buffalo, State University of New York Buffalo, NY United States; 2 Department of Epidemiology and Environmental Health School of Public Health and Health Professions University at Buffalo Buffalo, NY United States

**Keywords:** artificial intelligence, diabetes prevention, lifestyle interventions, systematic review, digital health, prediabetes

## Abstract

**Background:**

Artificial intelligence (AI)–driven lifestyle interventions are increasingly used for reducing diabetes risk. However, whether they produce clinical benefit beyond standard Diabetes Prevention Programs (DPPs) and whether effects vary by AI type, population, or delivery setting, remains uncertain.

**Objective:**

This study aimed to evaluate the effects of AI-driven lifestyle interventions on clinical, behavioral, and engagement outcomes among people with prediabetes or elevated diabetes risk and to assess whether effects differed by AI type, population, or delivery setting.

**Methods:**

PubMed, Embase, CINAHL, Web of Science, IEEE Xplore, ClinicalTrials.gov, and citation searches were completed on August 3, 2026, for English-language reports published from 2015. Eligible studies evaluated AI-driven digital lifestyle interventions among people with prediabetes or elevated diabetes risks, or healthy participants in feasibility studies. One reviewer (OIE) screened records and extracted data; a second reviewer (TN) independently verified exclusions and extraction. Study quality was assessed with National Heart, Lung, and Blood Institute tools appropriate to each design. Given substantial heterogeneity in designs, estimands, and outcome metrics, findings were synthesized narratively following the Synthesis Without Meta-Analysis guideline rather than pooled statistically.

**Results:**

Of 8032 records, 11 studies were included: 1 randomized, 1 nonrandomized comparative, 4 observational, and 5 uncontrolled pre-post studies. Seven evaluated AI lifestyle coaching systems, 2 predictive AI platforms, and 2 conversational AI chatbots. Ten of 11 studies used commercially developed platforms. Study quality was rated good for 5 studies, fair for 3, and poor for 3. The randomized controlled trial showed noninferiority of an AI-DPP versus human coach–led DPP for the combined 12-month outcome (23/183, 12.6% vs 23/185, 12.4%; risk difference 0.1%, 1-sided 95% CI –5.5%). Program initiation (171/183, 93.4% vs 153/185, 82.7%; risk difference 10.7%, 95% CI 4.2%-17.3%) and completion (117/183, 63.9% vs 93/185, 50.3%; risk difference 13.7%; 95% CI 3.6%-23.7%) were higher with AI, but this did not translate into superior clinical outcomes. The nonrandomized comparison found no advantage of AI-personalized messaging over static messaging in terms of BMI or physical activity. The remaining studies reported uncontrolled changes precluding causal inference. Evidence was insufficient to determine whether effects differed by population or delivery setting.

**Conclusions:**

One randomized trial supports noninferiority, not superiority, of a specific AI-driven DPP relative to human coaching; the remaining evidence was nonrandomized, predominantly commercially developed, and hypothesis-generating. To our knowledge, this is the first systematic review to stratify evidence on AI-driven diabetes prevention by AI mechanism—coaching, predictive, and conversational—and by outcome domain, extending prior scoping work that mapped apps without appraising study quality or comparative effectiveness. This structured mapping shows where evidence is the strongest (engagement and weight) and the weakest (glycemic causality and generalizability), giving health systems a concrete basis for deciding where AI-DPP tools may supplement, rather than replace, standard DPP delivery, pending independent, adequately powered randomized trials.

## Introduction

Prediabetes is a growing global health burden. In 2024, the estimated global prevalence was 12.0% for impaired glucose tolerance and 9.2% for impaired fasting glucose, with both projected to increase through 2050 [1]. Diagnostic thresholds differ in sensitivity and clinical usage across glycemic markers [2]. Prediabetes affects approximately one-third of US adults and is associated with increased risks of type 2 diabetes, cardiovascular disease, and stroke [3,4]. Annual progression to type 2 diabetes is estimated at 5% to 10%, and lifetime progression may reach 70% without intervention [5,6]. Lifestyle modification, including dietary change, physical activity, and weight loss, can delay or prevent progression [7].

The Diabetes Prevention Program (DPP) is a structured, evidence-based lifestyle intervention combining dietary change, physical activity, and behavioral coaching to prevent or delay progression to type 2 diabetes among high-risk adults [8]. The original DPP trial and its translations established that this approach can substantially reduce progression, and this evidence underpins the Centers for Disease Control and Prevention’s National DPP and similar programs now delivered internationally [9,10]. However, in an analysis of 44 low- and middle-income countries, fewer than half of high-risk individuals reported receiving fundamental prevention activities [11]. Structured prevention programs remain concentrated in a limited number of countries [12]. In the United States, access is uneven, particularly in rural and underserved communities, and participation may be constrained by program intensity, limited time, scheduling conflicts, and coach-participant cultural mismatch [13-15]. A recent scoping review confirmed that DPP reach remains lowest among the populations at greatest risk, reinforcing that the evidence-practice gap is one of access and sustained engagement rather than intervention efficacy [16]. These barriers highlight the need for scalable approaches that can extend prevention services without compromising effectiveness and equity.

Advances in artificial intelligence (AI), including adaptive algorithms, machine learning–based personalization, predictive analytics, and conversational agents, have enabled digital interventions that tailor recommendations, coaching intensity, and engagement strategies to individual users [17,18]. Integration of continuous glucose monitoring (CGM) with AI may also support personalized risk assessment and behavioral feedback [19], but the clinical effectiveness of these approaches remains uncertain. Several reviews have examined digital diabetes prevention interventions, but each leaves a distinct gap relevant here. A systematic review of digital health technologies for type 2 diabetes prevention found that just over half of included randomized trials achieved clinically significant outcomes, but AI-enabled and non-AI digital tools were evaluated together rather than distinguished by mechanism [20]. A broader systematic review of digital health interventions for type 2 diabetes prevention, structured around health system outcomes such as population health, patient experience, and cost, similarly pooled digital tools, including AI-based interventions, without isolating AI-specific effects or appraising evidence by AI type [21]. A 2025 scoping review by Lalani et al [17] mapped AI and machine learning apps across the prediabetes care continuum. That review did not systematically appraise study quality, evaluate comparative effectiveness against standard DPPs, or organize evidence by AI intervention type and outcome domain. Across this literature, no review has yet combined systematic quality appraisal with stratification of comparative evidence by AI mechanism and outcome domain, which is necessary to determine whether specific categories of AI intervention, rather than digital delivery in general, are associated with clinical benefit.

This review had three objectives: (1) to evaluate the effects of AI-driven lifestyle interventions on clinical, behavioral, and engagement outcomes; (2) to assess whether findings differed by AI intervention type; and (3) to assess whether findings differed by population or delivery setting.

## Methods

### Eligibility Criteria

This systematic review was reported in accordance with PRISMA (Preferred Reporting Items for Systematic Reviews and Meta-Analyses) 2020 [18], including the PRISMA 2020 for Abstracts checklist (Multimedia Appendix 1) and PRISMA-S (Preferred Reporting Items for Systematic Reviews and Meta-Analyses literature search extension) [22]. Eligibility criteria were established in advance based on the research question. Studies were eligible for inclusion if they (1) enrolled children, adolescents, or adults with prediabetes or elevated risk for type 2 diabetes, or healthy participants enrolled in feasibility or pilot studies; (2) evaluated a lifestyle intervention delivered through a mobile, web, or SMS text messaging platform with an AI-driven component; (3) included a comparator such as usual care, minimal or no intervention, a waitlist, a non-AI digital intervention, or a standard lifestyle program when the study design was comparative; (4) reported at least 1 clinical, behavioral, or engagement outcome; (5) used a randomized, nonrandomized comparative, observational, single-arm, feasibility, or pilot design; and (6) published a peer-reviewed English-language report from January 1, 2015, through August 3, 2026. Elevated diabetes risk was defined by impaired fasting glucose, impaired glucose tolerance, hemoglobin A_1c_ (HbA_1c_) of 5.7% to 6.4%, overweight or obesity (BMI at least 25 kg/m²), or a validated diabetes risk score.

For this review, an AI-driven intervention was defined as an intervention that used algorithmic personalization, adaptive goal setting, predictive modeling, machine learning–based tailoring, or a conversational system with individualized adaptation. Scripted, nonadaptive rule-based chatbots and fixed decision tree interventions without personalization, learning, or adaptation were excluded. Additional exclusions were qualitative-only studies, interventions delivered solely by human coaches without algorithmic personalization, interventions focused exclusively on treatment of established diabetes without a prevention or risk reduction objective, hardware-only technologies without a behavioral intervention, conference abstracts without full reports, protocols without results, editorials, commentaries, and reviews.

### Protocol and Registration

No formal review protocol was prepared, and the review was not prospectively registered. This reflects an exploratory synthesis rather than a prespecified confirmatory review.

### Information Sources

PubMed, Embase, CINAHL, Web of Science, and IEEE Xplore, and ClinicalTrials.gov were searched. Embase was searched via Elsevier platform, CINAHL via EBSCOhost, and Web of Science via the Clarivate Web of Science interface; PubMed, IEEE Xplore, and ClinicalTrials.gov were searched directly through their native web interfaces. Reference lists of included studies were examined for potentially eligible reports (backward citation searching), and forward citation searching was conducted in Google Scholar. No dissertation, preprint, or other gray literature databases were searched beyond ClinicalTrials.gov. Study authors, experts, and manufacturers were not contacted to identify additional studies or obtain unpublished data.

### Search Strategy

The searches were conducted on August 3, 2026, and included English-language reports published from January 1, 2015, through the search date (reflecting the increasing adoption of AI apps). The strategy combined controlled vocabulary and text words for 3 concepts: prediabetes or elevated diabetes risk, AI-driven digital health, and lifestyle behaviors or engagement relevant to diabetes prevention. The strategy was developed de novo rather than adapted or reused from a previously published review, and no validated study design search filter was applied, as none exists for this literature. The strategy was constructed and internally reviewed by the review team but was not peer reviewed by an independent librarian. The search was run once, on August 3, 2026, across all sources, and was not subsequently updated or rerun closer to submission. Records were exported to Covidence (Veritas Health Innovation), which was used for duplicate removal and screening [23]. The complete database strategies, platform-specific syntax, and source-specific record counts are provided in Multimedia Appendix 2, and the full PRISMA-S checklist [24], with “not applicable” items explained, is provided in Multimedia Appendix 3.

### Selection Process

One reviewer (OIE) screened all titles and abstracts and assessed all retrieved full-text reports against the eligibility criteria. A second reviewer (TN) independently verified all exclusion decisions. Disagreements were resolved through discussion, with adjudication by a third reviewer (RAF) when consensus could not be reached. No automation tool was used to determine eligibility. Detailed reason-level coding was retained for full-text exclusions but not for title and abstract exclusions.

### Data Collection Process

One reviewer (OIE) extracted data from all included studies using a standardized, piloted extraction form (Multimedia Appendix 4); a second reviewer (TN) independently verified all extracted data, with disagreements resolved by a third reviewer (RAF).

### Data Items

The extraction form (Multimedia Appendix 4) captured study design, country and setting, participant characteristics, sample size, intervention duration and delivery platform, AI functionality and origin (commercial, research-developed, or nonprofit or public), comparator, funding source, author-industry relationships when reported, and study findings. Outcomes were grouped as clinical (weight, BMI, HbA_1c_, fasting glucose, and CGM-derived metrics), behavioral (diet, physical activity, and sleep), or engagement (initiation, retention, use, session completion, adherence, and perceived usefulness). All reported outcome measures and time points relevant to these domains were sought. Unclear or unreported information was recorded as not reported; no values were imputed.

### Study Quality Assessment

Study quality was assessed using the National Heart, Lung, and Blood Institute (NHLBI) Study Quality Assessment Tools appropriate to each study design: controlled intervention studies, observational cohort and cross-sectional studies, and pre-post studies without a control group (Multimedia Appendix 5 [25-35]) [36]. The nonrandomized comparative study was evaluated with the controlled intervention studies tool, since its design included a concurrent, non–randomized control arm; items assessing random sequence generation and allocation concealment were scored “not applicable.” One reviewer (TN) completed each assessment, and a second reviewer (OIE) independently verified the ratings. Individual items were coded as “Yes,” “No,” “Cannot determine,” “Not reported,” or “Not applicable.” Because the NHLBI tools do not prescribe numeric cutoffs, overall ratings of “Good,” “Fair,” or “Poor” reflected qualitative judgment of the number and seriousness of design-specific limitations.

### Effect Measures

For the randomized trial, effect measures included between-group risk differences with 1-sided 95% CIs for the composite primary outcome and the weight loss threshold; glycemic, engagement, and physical activity outcomes were reported as between-group percentages or medians as presented by the investigators. Because program initiation and completion were not part of the trial’s prespecified noninferiority framework, 95% CIs for these risk differences were calculated by the review team using the normal approximation method for the difference of 2 independent proportions. For the nonrandomized comparative study, between-group mean differences, adjusted odds ratios, CIs, and *P* values were reported. For observational and uncontrolled pre-post studies, effect measures included within-group percentage or absolute change in weight, percentage-point changes in CGM metrics, changes in HbA_1c_ or fasting glucose, and percentage- or rate-based behavioral and engagement measures, as reported by each study.

### Synthesis Methods

Results were organized by study design and by 3 outcome domains: clinical, behavioral, and engagement. Findings were then stratified by investigator-defined AI intervention type: AI lifestyle coaching systems (adaptive goal setting and tailored feedback), predictive AI platforms (real-time or longitudinal data generating individualized predictions or recommendations), and conversational AI systems (chatbot interfaces emphasizing engagement). Variation by population and setting was explored descriptively across age, baseline metabolic risk, geographic location, and socioeconomic status. The randomized trial was prioritized in interpretation, followed by the nonrandomized comparative study; observational and uncontrolled pre-post findings were treated as descriptive and hypothesis-generating.

No meta-analysis was performed because the included studies evaluated fundamentally different designs and estimands and reported incompatible outcomes, including threshold-based risk differences, continuous percentage change, absolute change, CGM-derived metrics, and engagement measures, so a common standardized effect metric could not be calculated. Pooling randomized, nonrandomized comparative, observational, and uncontrolled pre-post evidence would have produced a summary estimate with unclear causal and clinical interpretation. Potential overlap among reports from the same commercial platforms further limited aggregation. No meta-analysis was performed; results were summarized narratively using the original metrics.

Weight and glycemic estimates are tabulated by study design in Multimedia Appendix 6 [25-35]. Ranges and medians were reported only when at least 2 studies within a design category used sufficiently comparable metrics. No statistical test of heterogeneity, meta-regression, or sensitivity analysis was conducted because no pooled estimate was calculated. The synthesis was reported using the Synthesis Without Meta-Analysis guideline (Multimedia Appendix 7).

### Reporting Bias Assessment

ClinicalTrials.gov was searched on August 3, 2026, using terms aligned with the database search concepts to identify completed, ongoing, or potentially unpublished eligible studies. The registry search did not add any included study. A formal statistical assessment of reporting bias was not performed because the number of studies was small, outcomes were heterogeneous, and many commercial evaluations were not prospectively registered.

### Certainty Assessment

A formal certainty-of-evidence framework, such as GRADE (Grading of Recommendations Assessment, Development, and Evaluation), was not prespecified or applied. The evidence could not be organized into sufficiently comparable intervention-comparator-outcome groupings for a formal domain-level certainty rating. Risk-of-bias assessments and study design were instead used to calibrate interpretation, and the absence of a formal certainty assessment is acknowledged as a limitation.

## Results

### Study Selection

The searches identified 8032 records: PubMed (n=1756), Embase (n=5172), CINAHL (n=493), Web of Science (n=300), IEEE Xplore (n=308), and backward or forward citation searching (n=3) (Figure 1). After 1732 duplicates were removed, 6300 records were screened by title and abstract, and 6272 records were excluded because they did not meet eligibility criteria. A total of 28 reports were sought and retrieved for full-text assessment. Overall, 17 were excluded: no trial results, trial registration, or protocol only (n=3); commentary (n=4); wrong intervention (n=2); wrong population (n=3); conference proceeding (n=4); and wrong study design (n=1). A total of 11 studies were included.

**Figure 1:**
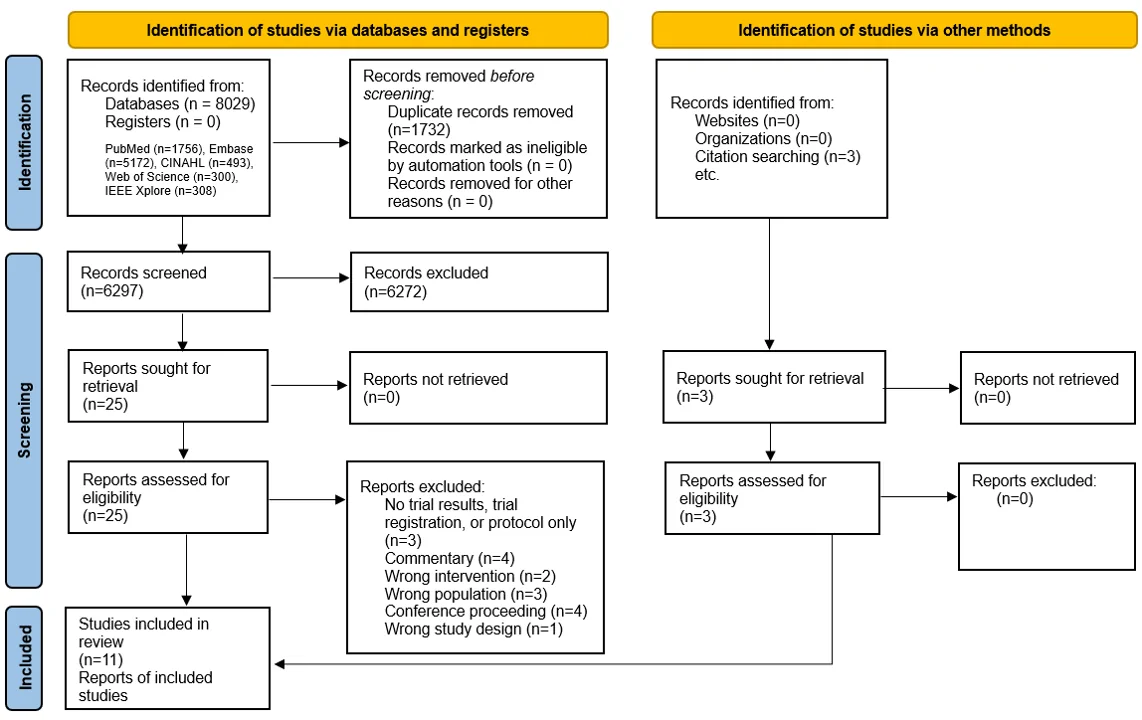
PRISMA (Preferred Reporting Items for Systematic Reviews and Meta-Analyses) 2020 flow diagram for the identification, screening, eligibility assessment, and inclusion of studies evaluating artificial intelligence–driven lifestyle interventions for reducing type 2 diabetes risk, searched through August 3, 2026.

### Study Characteristics

A total of 11 studies published from 2017 through 2025 evaluated AI-driven lifestyle interventions for reducing diabetes risk (Table 1). Ten studies were conducted in the United States, and 1 was conducted in rural India [25]. Populations were predominantly adults with prediabetes or elevated risk; 2 studies enrolled adolescents [26,27], and 1 study enrolled adults with no diabetes [25]. Study sample sizes ranged from 13 to 16,327 participants and summed to 30,9991 participant records, although overlap among related commercial platform reports precluded estimation of the number of unique participants. Follow-up ranged from 33 days to 12 months. The evidence base comprised 1 randomized clinical trial [28], 1 quasi-experimental nonrandomized comparative study [25], 4 observational studies [29-32], and 5 uncontrolled pre-post studies [26,27,33-35]. Seven studies evaluated AI lifestyle coaching systems [25,28-31,33,34], 2 evaluated predictive AI platforms [32,35], and 2 evaluated conversational AI chatbots [26,27]. Eight interventions were delivered primarily through mobile apps [28-35], 3 used SMS text messaging platforms [25-27], and 2 integrated CGM [32,35]. Ten studies evaluated commercially developed interventions; the Arogya World intervention was nonprofit-led [25]. No purely research-developed or publicly developed platforms were identified.

**Table 1:** Characteristics of 11 studies evaluating AI^a^-driven lifestyle interventions among children, adolescents, and adults with prediabetes or elevated diabetes risk in the United States and India (2017-2025).

Study	Population	AI type (company)	Delivery platform	Study design	Funding
Mathioudakis et al (2025) [28]	Adults with prediabetes, overweight, or obese (N=368)	AI lifestyle coaching system (Sweetch)^b^	Mobile health app	Randomized controlled trial	NIH (NIDDK)^c^
Chadwick et al (2025) [25]	Adults without diabetes aged 18-60 years (N =1048); 1082 enrolled and 1048 analyzed after exclusion of 34 control group noninitiators	AI lifestyle coaching system (Arogya)	WhatsApp messaging	Quasi-experimental nonrandomized comparative pre-post study	Google AI for Social Good Program, US Army Research Office Grant
Auster-Gussman et al (2022) [29]	Adults with prediabetes or overweight or obesity at high risk for type 2 diabetes (N=16,327)	AI lifestyle coaching system (Lark)^b^	Mobile health app	Observational cohort and cross-sectional study	Lark Health
Graham et al (2022) [30]	Adults with prediabetes or high risk for type 2 diabetes based on CDC^d^ risk score, with BMI ≥25 kg/m² (N=3933)	AI lifestyle coaching system (Lark)^b^	Mobile health app	Observational cohort and cross-sectional study	The authors reported no external financial support for the research, authorship, or publication; however, all listed authors were employees of Lark Health.
Graham et al (2023) [31]	Adults with prediabetes enrolled in a digital DPP^e^ (N=7266)	AI lifestyle coaching system (Lark)^b^	Mobile health app	Observational cohort and cross-sectional study	Funding not reported in the extracted study information; Lark platform was commercially developed.
Sapre et al (2024) [26]	Adolescents with prediabetes enrolled in a DPP (N=13)	Conversational AI chatbot (mPulse Mobile)^b^	SMS text messaging	Pre-post studies with no control group	NIH (NIDDK), Cigna Foundation
Stein and Brooks (2017) [34]	Adults at risk of diabetes, overweight, or obese (N=70)	AI lifestyle coaching system (Lark)^b^	Mobile health app	Pre-post studies with no control group	Lark Health
Veluvali et al (2025) [32]	Mixed glycemic status cohort, including participants with no diabetes (N=944); outcome-specific samples varied	Predictive AI platform (January AI)^b^	Mobile health app + CGM^f^ + wearable	Observational cohort and cross-sectional study	January AI
Zahedani et al (2023) [35]	Adults with prediabetes and diabetes (N=944)	Predictive AI platform (January AI)^b^	Mobile health app + CGM + wearable	Pre-post studies with no control group	January AI
Everett et al (2018) [33]	Mixed glycemic-status cohort (N=2217); glucose analysis (n=1066); outcome-specific samples varied	AI lifestyle coaching system (Sweetch)^b^	Mobile health app	Pre-post studies with no control group	Sweetch Health
Stephens et al (2019) [27]	Adolescents with obesity symptoms and metabolic risk (N = 23)	Conversational AI chatbot (X2AI)^b^	SMS text messaging	Pre-post studies with no control group	Funding not reported in the extracted study information; X2AI platform was commercially developed.

^a^AI: artificial intelligence.

^b^Commercially developed platform.

^c^NIH (NIDDK): National Institutes of Health (National Institute of Diabetes and Digestive and Kidney Diseases).

^d^CDC: Centers for Disease Control and Prevention.

^e^DPP: Diabetes Prevention Program.

^f^CGM: continuous glucose monitoring.

### Quality Assessment of Included Studies

Out of 11 studies, 5 were rated “Good,” 3 “Fair,” and 3 “Poor” (Table 2 and Multimedia Appendix 5). The randomized trial was rated “Good” [28], and the nonrandomized comparative study was rated “Fair” [25]. Among the 4 observational studies, Graham et al [30], Graham et al [31], and Veluvali et al [32] were rated “Good,” and Auster-Gussman et al [29] was rated “Fair.” Among the 5 uncontrolled pre-post studies, Everett et al [33] was rated “Good,” Zahedani et al [35] was rated “Fair,” and Sapre et al [26], Stein and Brooks [34], and Stephens et al [27] were rated “Poor.” Common limitations included lack of randomization or comparison groups, selection of highly engaged digital platform users, incomplete adjustment for confounding, attrition or outcome availability among subsets of enrolled participants, and short follow-up.

**Table 2:** Study-level quality ratings using NHLBI^a^ Study Quality Assessment Tools.

Study	Study design	NHLBI tool used	Overall rating
Mathioudakis et al (2025) [28]	Randomized controlled trial	Controlled Intervention Studies	Good
Chadwick et al (2025) [25]	Quasi-experimental nonrandomized comparative pre-post study	Controlled Intervention Studies	Fair
Auster-Gussman et al (2022) [29]	Observational cohort and cross-sectional study	Observational Cohort and Cross-Sectional Studies	Fair
Graham et al (2022) [30]	Observational cohort and cross-sectional study	Observational Cohort and Cross-Sectional Studies	Good
Veluvali et al (2025) [32]	Observational cohort and cross-sectional study	Observational Cohort and Cross-Sectional Studies	Good
Graham et al (2023) [31]	Observational cohort and cross-sectional study	Observational Cohort and Cross-Sectional Studies	Good
Sapre et al (2024) [26]	Uncontrolled pre-post study	Before-After (Pre-Post) Studies	Poor
Stein and Brooks 2017 [34]	Uncontrolled pre-post study	Before-After (Pre-Post) Studies	Poor
Zahedani et al (2023) [35]	Uncontrolled pre-post study	Before-After (Pre-Post) Studies	Fair
Everett et al (2018) [33]	Uncontrolled pre-post study	Before-After (Pre-Post) Studies	Good
Stephens et al (2019) [27]	Uncontrolled pre-post study	Before-After (Pre-Post) Studies	Poor

^a^NHLBI: National Heart, Lung, and Blood Institute.

### Results of Individual Studies

The randomized trial by Mathioudakis et al [28] provided the strongest comparative evidence (Table 3 and Multimedia Appendix 6). For the combined target of at least 4% weight loss and at least 150 minutes per week of physical activity, 12.6% (23/183) of AI-DPP participants and 12.4% (23/185) of human coach–led DPP participants met the outcome (risk difference 0.1%; 1-sided 95% CI –5.5%), supporting noninferiority. For at least 5% weight loss, the corresponding proportions were 16.9% (31/183) and 20.0% (37/185) (risk difference –3.1%; 1-sided 95% CI –9.7%), with the lower bound remaining within the prespecified –15 percentage point noninferiority margin. Among participants with prediabetes-range HbA_1c_ at baseline, in each arm, 26.9% (35/130) achieved at least 0.2% point reduction. Program initiation (171/183, 93.4% vs 152/185, 82.7%; risk difference 10.7%, 95% CI 4.2%-17.3%) and completion (117/183, 63.9% vs 93/185, 50.3%; risk difference 13.7%, 95% CI 3.6%-23.7%) were higher in the AI-DPP arm, but these engagement differences did not translate into superior clinical outcomes.

In the quasi-experimental study by Chadwick et al [25], AI-personalized messaging did not improve BMI compared with static messaging (between-group change 0.0 kg/m², 95% CI –0.6 to 0.5; *P*=.95) and did not increase the odds of achieving at least 30 minutes per day of physical activity (adjusted odds ratio 1.0, 95% CI 0.7-1.3; *P*=.74). Overall program completion was 96.9% (1048/1082). Because allocation was not randomized, residual confounding and selection bias remain possible.

The 4 observational studies reported uncontrolled weight changes. Across the 2 reports using percentage change, the median was 4.53% (range 4.1%-4.96%) [30,31]. Across the 2 reports using absolute change, the median was 2.8 kg (range 1.5-4.1 kg) [29,32]. Behavioral and engagement outcomes were not reported for these studies.

**Table 3:** Clinical, behavioral, and engagement outcomes for the 11 included studies, stratified by study design (1 randomized trial, 1 nonrandomized comparative study, 4 observational studies, and 5 before-after studies)^a^.

Study		Intervention duration	Intervention frequency or dose	Clinical outcome	Behavioral outcome	Engagement outcome
**Randomized controlled trial**						
	Mathioudakis et al (2025) [28]	12 months	AI-DPP^b^: continuous app access with daily personalized push notifications Human-led DPP: 16 weekly sessions (core phase), then biweekly to monthly sessions	Weight (at 12 months): (1) 12.6% (23/183) in AI-DPP vs 12.4% (23/185) in human coach–led DPP^c^ achieved ≥4% weight loss plus ≥150 minutes physical activity per week (RD^d^ 0.1%, 1-sided 95% CI –5.5%); (2) 16.9% (31/183) in AI-DPP vs 20.0% (37/185) in human coach–led DPP achieved ≥5% weight loss (RD –3.1%, 1-sided 95% CI –9.7%), a numerical difference favoring the human-led DPP; exploratory, not formally hypothesis-tested. Glycemic control: 26.9% (35/130) in AI-DPP vs 26.9% (35/130) in human coach–led DPP achieved ≥0.2 percentage points reduction in HbA_1c_^e^ (RD 0.0%,1-sided 95% CI –9.1%); among participants with prediabetes-range HbA_1c_ at baseline only (n=130 per group).	Physical activity (at 12 months): 210.5 minutes/week, IQR 134.5-344 in the AI-DPP vs 174.4 minutes, IQR 73.9-321.3 in the human coach–led DPP	Program initiation: 93.4% (171/183) in AI-DPP vs 82.7% (153/185) in human coach–led DPP (RD 10.7%, 95% CI 4.2%-17.3%; *P*=.001). Program completion: 63.9% (117/183) in AI-DPP vs 50.3% (93/185) in human coach–led DPP (RD 13.7%, 95% CI 3.6%-23.7%; *P*=.008).
**Quasi-experimental nonrandomized comparative study**						
	Chadwick et al (2025) [25]	6 months	Two WhatsApp messages per week (AI-personalized or static nonpersonalized) Messages delivered twice weekly over 6 months	Weight: No difference in BMI between AI^f^-personalized messaging and nonpersonalized messaging (–0.0 kg/m², 95% CI –0.6 to 0.5; *P*=.95)	Physical activity: Similar odds of achieving at least 30 minutes/day in AI-personalized vs static messaging (aOR^g^ 1.0, 95% CI 0.7-1.3; *P*=.74).	Program completion: 96.9% (1048/1082) overall; no significant between-group difference reported.
**Observational cohort and cross-sectional studies**						
	Auster-Gussman et al (2022) [29]	12 months	On-demand AI coaching available 24/7; 26 educational lessons; weekly weigh-ins encouraged via connected digital scale	Weight: 81.8% lost weight while enrolled; average weight loss 4.1 kg (SE=0.05 kg)^h^.	Not reported	Not reported
	Graham et al (2022) [30]	12 months	On-demand AI coaching available 24/7; 26 educational lessons; CDC^i^ qualifying criteria required ≥4 lessons over ≥9 months	Weight: 4.1% weight loss at 12 months among those providing CDC-required 12-month weight (414/3933, 10.53%); 5.3% among CDC qualifiers vs 3.3% among nonqualifiers (adjusted means; *P*=.015)	Not reported	Not reported
	Graham et al (2023) [31]	12 months	On-demand AI coaching available 24/7 via mobile app; CDC-recognized DPP curriculum; 26 educational lessons	Weight: 4.55%-4.96% mean weight loss across people living in hard-to-reach and underserved areas.^j^	Not reported	Not reported
	Veluvali et al (2025) [32]	33 days	Continuous CGM^k^-integrated mobile app with daily personalized feedback; wearable device data integrated	Weight: 3.3 lb (1.5 kg) reduction from baseline over 33 days. Glycemic control: TIR^l^ increased from 74.7% to 85.5% in healthy users (*P*<.0001).	Not reported	Not reported
**Uncontrolled pre-post studies**						
	Sapre et al (2024) [26]	12 weeks	Average of 2 automated SMS text messages per day delivered at 9 AM, 3 PM, 6 PM, or 9 PM depending on message type. Feasibility and acceptability process study: no clinical or behavioral outcomes assessed.	Not reported	Not reported	Program completion: 69% engaged at completion. Engagement: 46% highly responsive.
	Stein and Brooks (2017) [34]	Average 15 weeks (SD 1.0)	On-demand text-based AI coaching; average 103 sessions per user over the program period; no fixed frequency prescribed.	Weight: 2.38% weight loss from baseline (SEM 0.69%, 95% CI –3.75% to –1.00%)^m^	Diet: Healthy meals increased by 31%; unhealthy meals decreased by 54%^n^	Program completion: Users engaged for an average of 15 weeks (SD 1.0); average 103 sessions per user
	Zahedani et al (2023) [35]	12 weeks	Continuous CGM-integrated mobile app with daily personalized feedback; wearable device data integrated	Weight: 1.5% (healthy) and 2.3% (prediabetes) weight loss from baseline at 12 weeks. Glycemic control: TIR increased by 6.2% in those with prediabetes; hyperglycemic events reduced by 24%-42% depending on threshold.	Physical activity: Physical activity increased modestly, with time spent at heart rate >110 bpm rising from 28.8 to 32.2 minutes/day (*P*=.008)	Not reported
	Everett et al (2018) [33]	90 days (approximately 3 months)	Continuous on-demand AI coaching via mobile app available 24/7; connected digital body weight scale provided for regular weigh-ins; push notification frequency not explicitly quantified	Weight: 1.6-kg reduction from baseline (SD 2.5; *P*<.001). Glycemic control: –0.1% HbA1c change from baseline (IQR –0.2% to 0.1%; *P*=.04); –1 mg/dL fasting glucose from baseline (*P*=.59, not significant)	Physical activity: +2.8 MET^o^-hours/week from baseline (SD 6.8; *P*=.02)	Program completion: 86% participants retained
	Stephens et al (2019) [27]	Approximately 10-12 weeks (varied by recruitment timing within a 6-month rolling recruitment period)	On-demand 24/7 SMS chatbot; no fixed message frequency; mix of Tess-initiated (73.6%) and patient-initiated (26.4%) conversations. Feasibility study: no clinical or behavioral outcomes assessed.	Not reported	Not reported	Engagement: 81% reported progress toward goals; 96% reported usefulness; 4123 messages exchanged^p^

^a^Sapre et al [26] and Stephens et al [27] were feasibility studies; all reported figures are descriptive pilot metrics without formal hypothesis testing and cannot be interpreted as evidence of intervention effectiveness. For Mathioudakis et al [28], formal noninferiority testing applies to the primary composite outcome. Individual components are exploratory. The HbA_1c_ reduction component applies to participants with prediabetes-range HbA_1c_ at baseline (n=130 per arm). A reported 1-sided 95% confidence bound is the lower bound and not a 2-sided interval.

^b^AI-DPP: artificial intelligence–Diabetes Prevention Program.

^c^DPP: Diabetes Prevention Program.

^d^RD: risk difference.

^e^HbA_1c_: hemoglobin A_1c_.

^f^AI: artificial intelligence.

^g^aOR: adjusted odds ratio.

^h^Reported as a descriptive characteristic within a reach and demographics study; no formal hypothesis testing of weight outcomes was conducted and no comparator group was included.

^i^CDC: Centers for Disease Control and Prevention.

^j^No formal between-group hypothesis test reported for geographic subgroups.

^k^CGM: continuous glucose monitoring.

^l^TIR: time in range.

^m^Descriptive only; no formal hypothesis test reported for the primary weight outcome; no comparator group.

^n^Descriptive only; no *P* values reported for dietary outcomes.

^o^MET: metabolic equivalent of task.

^p^All figures are descriptive metrics from a feasibility study (N=23); no formal hypothesis testing was conducted.

The 5 uncontrolled pre-post studies reported within-group changes without a concurrent comparison group. Two short-duration CGM-integrated studies (1 observational, Veluvali et al [32], and 1 uncontrolled pre-post, Zahedani et al [35]) reported increases in time in range and reductions in hyperglycemic events. These glycemic findings are hypothesis-generating because comparison groups were absent and the observed changes cannot be separated from regression to the mean, monitoring-related behavior change, or other uncontrolled factors. In the uncontrolled pre-post study by Everett et al [33], participants had a mean weight change of –1.6 kg, a median HbA_1c_ change of –0.1 percentage point, and a mean physical activity change of 2.8 metabolic equivalent of task hours per week. Across the 2 reports using percentage weight change, the median was 2.34% (range 2.3%-2.38%) [34,35]. Stein and Brooks [34] also reported changes in meal patterns. The 2 adolescent chatbot studies (Sapre et al [26] and Stephens et al [27]) primarily reported feasibility, responsiveness, perceived usefulness, or goal progress rather than clinical effectiveness. These nonrandomized findings do not permit causal inference.

### Result of Syntheses by AI Type

As shown in Table 4, AI lifestyle coaching systems were the most frequently evaluated category (7 studies) and included the only randomized trial [25,28-31,33,34]. That trial supported noninferiority of 1 AI-DPP to a human coach–led DPP and found higher initiation and completion without superior clinical outcomes [28]. The remaining coaching system studies reported nonrandomized weight, behavioral, or engagement findings. Two predictive AI platforms integrated CGM and reported short-term within-group glycemic changes but neither used a concurrent comparison group, so causal attribution to the AI or CGM component is not possible [32,35]. Evidence for conversational AI chatbots was limited to 2 small feasibility or process studies focused primarily on engagement and perceived usefulness [26,27].

**Table 4:** Narrative synthesis of clinical, behavioral, and engagement findings from 11 studies of artificial intelligence (AI)–driven lifestyle interventions for reducing diabetes risk, stratified by AI type, population characteristics, and delivery setting, United States and India (2017-2025).

Domain			Clinical outcomes		Behavioral outcomes		Engagement outcomes		Interpretation
**AI^a^ type**									
	AI Lifestyle Coaching Systems (n=7)	One randomized trial supported noninferiority of a specific AI-DPP^b^; nonrandomized studies reported modest weight changes.		The RCT^c^ reported higher median physical activity in the AI arm; nonrandomized studies reported diet or activity changes.		Higher initiation and completion in the RCT; engagement measures varied across nonrandomized studies.		Most frequently studied AI type, but only 1 randomized comparison.	
	Predictive AI Platforms (n=2)	Two short, uncontrolled CGM^d^-integrated studies reported weight and glycemic changes; causal interpretation is not possible.		Uncontrolled studies reported changes in meal timing and dietary composition, or a small increase in time with heart rate above 110 beats/minute.		Not reported.		Hypothesis-generating only; effects cannot be separated from monitoring, regression to the mean, or other uncontrolled factors.	
	Conversational AI Chatbot (n=2)	Not reported.		Not reported.		Feasibility studies reported responsiveness, goal progress, or perceived usefulness.		Evidence limited to small feasibility or process studies.	
**Population and setting**									
	Age (Adolescent vs Adult)	Not comparable across groups.		Not comparable across groups.		Adolescent studies reported engagement only.		Major evidence gap; no cross-age comparisons possible.	
	Baseline Metabolic Risk	One uncontrolled study reported larger time-in-range changes in selected baseline-glycemia and baseline-TIR^e^ subgroups.		NR^f^		NR		Suggestive only; no randomized subgroup comparison.	
	Geographic Setting	One observational study reported similar rural and nonrural weight changes; 1 nonrandomized study in India found no advantage of AI-personalized messaging.		NR		NR		Insufficient evidence to determine geographic effect modification.	
	Socioeconomic Status	Not reported by socioeconomic status stratum		NR		NR		Effect heterogeneity by socioeconomic status remains unknown.	

^a^AI: artificial intelligence.

^b^AI-DPP: artificial intelligence–Diabetes Prevention Program.

^c^RCT: randomized controlled trial.

^d^CGM: continuous glucose monitoring.

^e^TIR: time in range.

^f^NR: not reported.

### Result of Syntheses by Populations and Delivery Settings

Evidence was insufficient to determine whether effects differed by populations or settings (Table 4). The 2 adolescent studies reported primarily engagement outcomes and could not be compared with adult studies [26,27]. One uncontrolled study reported larger changes in time in range among participants with poorer baseline glycemic status, but no randomized subgroup comparison was performed [35]. One observational study reported similar changes in weight across rural and nonrural subgroups [31], whereas a nonrandomized study conducted in rural India found no advantage of AI-personalized messaging over static messaging in terms of BMI or physical activity [25]. No study reported adequately powered income-stratified or socioeconomic status–stratified treatment effects.

### Reporting Biases

The ClinicalTrials.gov search did not identify any completed study. No formal reporting bias judgment was made because the evidence base was small, outcomes were heterogeneous, and registration was uncommon among commercial platform evaluations.

### Certainty of Evidence

Certainty of evidence was not formally rated. The predominance of nonrandomized designs, limited replication, inconsistent outcome reporting, and potential commercial sponsorship bias reduced confidence in conclusions beyond the single randomized comparison.

## Discussion

### Principal Findings

This systematic review addressed 3 objectives: evaluating the clinical, behavioral, and engagement effects of AI-driven lifestyle interventions; determining whether effects differed by AI intervention type; and assessing whether effects differed by population or delivery setting. For the first objective, the evidence base was small and methodologically heterogeneous. The only randomized controlled trial supported noninferiority of 1 AI-DPP to a human coach–led DPP, but greater engagement did not produce superior clinical outcomes. A nonrandomized comparative study found no benefit of AI-personalized messaging over static messaging in terms of BMI or physical activity, and observational and uncontrolled pre-post studies reported weight, glycemic, behavioral, and engagement changes that do not support causal attribution. For the second objective, findings differed descriptively by AI type: coaching systems had the broadest evidence base, predictive platforms reported short-term glycemic changes only in uncontrolled settings, and conversational chatbot evidence remained limited to small feasibility studies. For the third objective, evidence was insufficient to determine whether effects differed by age, socioeconomic status, geographic setting, or baseline metabolic risk.

These findings extend the scoping review by Lalani et al [17], which described the breadth of AI apps in prediabetes but did not systematically appraise quality or organize effectiveness evidence by AI type and outcome domain. The current review’s single randomized controlled trial suggests that engagement and clinical effectiveness should, at least provisionally, be interpreted separately: higher initiation and completion in the AI arm did not confer superior clinical benefit [28]. This warrants revisiting as more evidence emerges, since a systematic review and meta-analysis of digital mental health interventions found the opposite, that greater engagement was generally associated with better outcomes [37]. Engagement’s value as a proxy for effectiveness may therefore be context-specific rather than fixed, and larger, adequately powered trials are needed before the dissociation seen here is treated as more than a feature of this 1 trial. Coaching systems have the broadest evidence base, but most supporting studies are nonrandomized. Predictive platforms have reported short-term CGM changes, but these observations cannot be distinguished from monitoring effects, regression to the mean, or concurrent behavior change [32,35]. Chatbot evidence remains limited to small feasibility studies [26,27]. AI-driven interventions may offer flexible and scalable delivery, but they also introduce barriers related to digital literacy, internet and device access, affordability, language, and cultural fit [38,39]. Health systems may reasonably evaluate these tools as adjuncts to established prevention services, particularly when implementation includes equity monitoring and human support, but the evidence does not support replacing standard DPPs or assuming that increased engagement will improve clinical outcomes.

These findings carry direct implications for how health systems deploy AI-driven tools alongside standard DPP delivery. Because access to in-person DPP remains uneven, particularly in rural and underserved communities, and participation is often constrained by program intensity, scheduling conflicts, and coach-participant cultural mismatch [13-15], and because DPP reach remains lowest among the populations at greatest risk [16], AI-driven platforms offer a plausible route to extend reach where implementation barriers, not intervention efficacy, are the limiting factor. The randomized evidence here supports this reach argument more than a clinical superiority argument: the AI-DPP achieved meaningfully higher initiation and completion than human coaching, which matters practically for programs struggling to enroll and retain high-risk populations, although it did not outperform coaching on clinical end points. For health systems and payers, this suggests that AI-DPP tools are best positioned as a scalable engagement layer that extends the reach of the Centers for Disease Control and Prevention’s National DPP model [9,10], a way to channel more at-risk individuals into some form of structured prevention programming, rather than as a substitute expected to outperform coach-led care on weight, glycemic, or activity outcomes. This distinction matters for real-world deployment decisions: because structured prevention programs remain concentrated in a limited number of countries and settings [12], a program evaluating whether to adopt an AI-DPP tool to reach a larger population should weigh it primarily against the counterfactual of no program participation at all, where higher initiation and completion are likely to translate into more people receiving some prevention benefit, rather than against human coaching alone. At the same time, because 10 of 11 included studies evaluated commercially developed platforms with limited independent replication, health systems should treat vendor-reported engagement and weight outcomes cautiously until independent, adequately powered trials, ideally across diverse populations and settings, confirm generalizability beyond the single existing randomized comparison.

### Limitations

This review has limitations at both the evidence and review process levels. Most included studies were observational or uncontrolled and were susceptible to selection bias, confounding, regression to the mean, and outcome availability among selected subsets [40,41]. Follow-up was often short, and outcomes were measured and reported inconsistently. Most interventions were commercially developed, and several studies reported direct industry funding, employment, or authorship involvement. Because the NHLBI tools do not incorporate funding source, this potential favorable outcome and selective reporting bias was considered separately in interpretation and summarized in Table 2 [42]. Participants’ overlap across reports from the same commercial platforms could not be excluded. Restriction to English-language publications may have introduced language bias and reduced representation of evidence from non-Western settings. The initial screening and data extraction were performed by 1 reviewer (OIE) and verified by a second reviewer (TN) rather than being conducted independently in duplicate. The review was not prospectively registered, no formal protocol was prepared, and no formal certainty assessment was conducted. Meta-analysis was not performed because designs, estimands, and outcome metrics were not sufficiently comparable; consequently, differences across AI types or populations were descriptive rather than statistically tested. Rapid development of AI systems also means that findings may not generalize to newer large language model–based tools [43,44]. Future studies should use adequately powered randomized designs, prespecified subgroup analyses, standardized clinical and engagement outcomes, longer follow-up, transparent reporting of AI methods and commercial relationships, and independent evaluation in low- and middle-income countries and noncommercial settings.

### Conclusions

The evidence does not establish clinical superiority of AI-driven lifestyle interventions over standard diabetes prevention care. One randomized trial supports noninferiority of a specific AI-DPP at 12 months; other findings are predominantly nonrandomized, commercially developed, and hypothesis-generating. To our knowledge, this is the first systematic review to stratify AI-driven diabetes prevention evidence by AI mechanism (coaching, predictive, and conversational) and by outcome domain, extending prior scoping work that mapped the breadth of AI apps in this space without appraising study quality or comparative effectiveness against standard DPPs. This design- and mechanism-stratified synthesis makes explicit where evidence is strongest (engagement and weight) and weakest (causally interpretable glycemic, subgroup, and generalizability effects), a distinction that broader digital health reviews pooling AI and non-AI interventions together have not drawn. For health systems and payers weighing AI-DPP adoption, this mapping offers a concrete, evidence-based basis for treating these tools as adjuncts that may extend reach and engagement, rather than substitutes expected to match or exceed standard DPP outcomes, while monitoring for equity effects as platforms scale. Effectiveness, sustainability, equity, and cost-effectiveness of AI-driven lifestyle interventions require independent, adequately powered randomized trials before broader adoption is warranted.

## Data Availability

All data generated or analyzed during this study are included in this published article and in the Multimedia Appendices 1-8.

## Appendix

Multimedia Appendix 1 PRISMA 2020 for abstract checklist.

Multimedia Appendix 2 Search strategy.

Multimedia Appendix 3 PRISMA-S checklist (search reporting).

Multimedia Appendix 4 Data extraction form.

Multimedia Appendix 5 Methodological quality.

Multimedia Appendix 6 Weight loss and glycemic effect estimates by study design.

Multimedia Appendix 7 SWiM (Synthesis Without Meta-Analysis) checklist (synthesis without meta-analysis).

Multimedia Appendix 8 PRISMA checklist.

## Abbreviations

AI: artificial intelligence
CGM: continuous glucose monitoring
DPP: Diabetes Prevention Program
GRADE: Grading of Recommendations Assessment, Development, and Evaluation
HbA1c: hemoglobin A1c
NHLBI: National Heart, Lung, and Blood Institute
PRISMA: Preferred Reporting Items for Systematic Reviews and Meta-Analyses
PRISMA-S: Preferred Reporting Items for Systematic Reviews and Meta-Analyses literature search extension
