# Supplementary material for: AI-Driven Lifestyle Interventions for Reducing Diabetes Risk: Systematic Review
Source: J Med Internet Res. 2026 Oct 15;28:e104940. doi: 10.2196/104940 (PMC13720412; doi:10.2196/104940)
Supplement: Supplementary file 2 [file jmir_v28i1e104940_app2.docx]

## Multimedia Appendix 2. Search Strategy

A. PubMed

| No | Core concept | Search |
| --- | --- | --- |
| 1 | Prediabetes | ("Prediabetic State"[Mesh] OR prediabetes[tiab] OR pre-diabetes[tiab] OR "diabetes prevention"[tiab] OR "type 2 diabetes prevention"[tiab] OR "impaired glucose"[tiab] OR "impaired glucose tolerance"[tiab] OR "impaired fasting glucose"[tiab] OR IFG[tiab] OR IGT[tiab] OR dysglycemia[tiab] OR "metabolic health"[tiab] OR "metabolic disease"[tiab] OR "youth-onset type 2 diabetes"[tiab] OR OR "elevated diabetes risk"[tiab]) |
| 2 | AI-driven digital health | ("Artificial Intelligence"[MeSH] OR "Machine Learning"[MeSH] OR "Deep Learning"[MeSH] OR "Algorithms"[MeSH] OR "Wearable Electronic Devices"[MeSH] OR "Blood Glucose Self-Monitoring"[MeSH] OR "Text Messaging"[MeSH] OR "Precision Medicine"[MeSH] OR AI[tiab] OR "machine learning"[tiab] OR "deep learning"[tiab] OR algorithm*[tiab] OR chatbot*[tiab] OR "conversational agent*"[tiab] OR "conversational artificial intelligence"[tiab] OR "natural language processing"[tiab] OR "computer-assisted"[tiab] OR "digital health"[tiab] OR "eHealth"[tiab] OR "mobile health"[tiab] OR mHealth[tiab] OR "mobile app*"[tiab] OR "application"[tiab] OR "e-learning"[tiab] OR "web-based intervention*"[tiab] OR "text messag*"[tiab] OR SMS[tiab] OR "continuous glucose monitoring"[tiab] OR CGM[tiab] OR "digital twin"[tiab] OR "predictive analytics"[tiab] OR "predictive model*"[tiab] OR "personalization algorithm*"[tiab] OR "adaptive intervention*"[tiab] OR "tailored feedback"[tiab] OR "fully automated"[tiab] OR wearable*[tiab]) |
| 3 | Lifestyle behaviors related to diabetes prevention | ("Life Style"[Mesh] OR "Exercise"[Mesh] OR "Diet, Healthy"[Mesh] OR "Weight Loss"[Mesh] OR "Patient Participation"[Mesh] OR "Health Services Accessibility"[Mesh] OR "Rural Health Services"[Mesh] OR lifestyle[tiab] OR "physical activity"[tiab] OR exercise[tiab] OR diet*[tiab] OR "weight loss"[tiab] OR nutrition[tiab] OR "Obesity"[Mesh] OR obesity[tiab] OR overweight[tiab] OR "abdominal obesity"[tiab] OR BMI[tiab] OR "time in range"[tiab] OR "glucose management indicator"[tiab] OR "patient engagement"[tiab] OR "user engagement"[tiab] OR "program completion"[tiab] OR "program initiation"[tiab] OR retention[tiab] OR reach[tiab] OR "health professional shortage area*"[tiab] OR HPSA[tiab] OR rural[tiab] OR underserved[tiab] OR "hard-to-reach"[tiab]) |

| 4 | 1 AND 2 AND 3 | (#1 AND #2 AND #3) AND (english[lang]) AND ("2015/01/01"[Date -  Publication]: "3000"[Date - Publication]) |
| --- | --- | --- |

1756 files imported on 8/3/2026

B. Embase

| No | Core concept | Search |
| --- | --- | --- |
| 1 | Prediabetes | ('prediabetes'/exp OR 'impaired glucose tolerance'/exp OR 'impaired fasting glucose'/exp OR prediabetes:ti,ab,kw OR 'pre-diabetes':ti,ab,kw OR 'diabetes prevention':ti,ab,kw OR 'type 2 diabetes prevention':ti,ab,kw OR 'impaired glucose':ti,ab,kw OR 'impaired glucose tolerance':ti,ab,kw OR 'impaired fasting glucose':ti,ab,kw OR IFG:ti,ab,kw OR IGT:ti,ab,kw OR dysglycemia:ti,ab,kw OR 'metabolic health':ti,ab,kw OR 'metabolic disease':ti,ab,kw OR 'youth-onset type 2 diabetes':ti,ab,kw OR 'adolescents with prediabetes':ti,ab,kw OR 'at risk for type 2 diabetes':ti,ab,kw OR 'elevated diabetes risk':ti,ab,kw) |
| 2 | AI-driven digital health | ('artificial intelligence'/exp OR 'machine learning'/exp OR 'deep learning'/exp OR 'algorithm'/exp OR 'wearable computer'/exp OR 'blood glucose monitoring'/exp OR 'text messaging'/exp OR 'precision medicine'/exp OR 'natural language processing'/exp OR 'chatbot'/exp OR 'digital health'/exp OR 'ehealth'/exp OR 'mobile health'/exp OR 'mobile application'/exp OR 'web based intervention'/exp OR AI:ti,ab,kw OR 'machine learning':ti,ab,kw OR 'deep learning':ti,ab,kw OR algorithm*:ti,ab,kw OR chatbot*:ti,ab,kw OR 'conversational agent*':ti,ab,kw OR 'conversational artificial intelligence':ti,ab,kw OR 'natural language processing':ti,ab,kw OR 'computer assisted':ti,ab,kw OR 'digital health':ti,ab,kw OR ehealth:ti,ab,kw OR 'mobile health':ti,ab,kw OR mhealth:ti,ab,kw OR 'mobile app*':ti,ab,kw OR application:ti,ab,kw OR 'e-learning':ti,ab,kw OR 'web-based intervention*':ti,ab,kw OR 'text messag*':ti,ab,kw OR SMS:ti,ab,kw OR 'continuous glucose monitoring':ti,ab,kw OR CGM:ti,ab,kw OR 'digital twin':ti,ab,kw OR 'predictive analytics':ti,ab,kw OR 'predictive model*':ti,ab,kw OR 'personalization algorithm*':ti,ab,kw OR 'adaptive intervention*':ti,ab,kw OR 'tailored feedback':ti,ab,kw OR 'fully automated':ti,ab,kw OR wearable*:ti,ab,kw) |
| 4 | Lifestyle behaviors related to | ('lifestyle'/exp OR 'exercise'/exp OR 'healthy diet'/exp OR 'weight reduction'/exp OR 'nutrition'/exp OR 'obesity'/exp OR 'overweight'/exp OR 'patient participation'/exp OR 'health care  access'/exp OR 'rural health care'/exp OR lifestyle:ti,ab,kw OR |

|  | diabetes prevention | 'physical activity':ti,ab,kw OR exercise:ti,ab,kw OR diet*:ti,ab,kw OR 'weight loss':ti,ab,kw OR nutrition:ti,ab,kw OR obesity:ti,ab,kw OR overweight:ti,ab,kw OR 'abdominal obesity':ti,ab,kw OR BMI:ti,ab,kw OR 'time in range':ti,ab,kw OR 'glucose management indicator':ti,ab,kw OR 'patient engagement':ti,ab,kw OR 'user engagement':ti,ab,kw OR 'program completion':ti,ab,kw OR 'program initiation':ti,ab,kw OR retention:ti,ab,kw OR reach:ti,ab,kw OR 'health professional shortage area*':ti,ab,kw OR HPSA:ti,ab,kw OR rural:ti,ab,kw OR underserved:ti,ab,kw OR 'hard-to-reach':ti,ab,kw) |
| --- | --- | --- |
| 5 | 1 AND 2 AND 3 | #1 AND #2 AND #3 AND [english]/lim AND [2015-2026]/py |

5172 files imported 8/3/2026

C. CINAHL

| No | Core concept | Search |
| --- | --- | --- |
| 1 | Prediabetes | (MH "Prediabetic State+" OR MH "Glucose Intolerance+" OR MH "Impaired Fasting Glucose" OR TI (prediabetes OR pre-diabetes OR "diabetes prevention" OR "type 2 diabetes prevention" OR "impaired glucose" OR "impaired glucose tolerance" OR "impaired fasting glucose" OR IFG OR IGT OR dysglycemia OR "metabolic health" OR "metabolic disease" OR "youth-onset type 2 diabetes" OR "adolescents with prediabetes" OR "at risk for type 2 diabetes" OR "elevated diabetes risk") OR AB (prediabetes OR pre-diabetes OR "diabetes prevention" OR "type 2 diabetes prevention" OR "impaired glucose" OR "impaired glucose tolerance" OR "impaired fasting glucose" OR IFG OR IGT OR dysglycemia OR "metabolic health" OR "metabolic disease" OR "youth-onset type 2 diabetes" OR "adolescents with prediabetes" OR "at risk for type 2 diabetes" OR "elevated diabetes risk")) |
| 2 | AI-driven digital health | (MH "Artificial Intelligence+" OR MH "Machine Learning" OR MH "Neural Networks (Computer)+" OR MH "Natural Language Processing" OR MH "Chatbots" OR MH "Mobile Applications" OR MH "Mobile Health" OR MH "Telehealth+" OR MH "Computer-Assisted Instruction+" OR MH "Text Messaging" OR MH "Wearable Electronic Devices" OR MH "Blood Glucose Self-Monitoring" OR MH "Precision Medicine" OR TI (AI OR "machine learning" OR "deep learning" OR algorithm* OR chatbot* OR "conversational agent*" OR "conversational artificial intelligence" OR "natural language processing" OR "computer-assisted" OR "digital health"  OR eHealth OR "mobile health" OR mHealth OR "mobile app*" OR |

|  |  | application OR "e-learning" OR "web-based intervention*" OR "text messag*" OR SMS OR "continuous glucose monitoring" OR CGM OR "digital twin" OR "predictive analytics" OR "predictive model*" OR "personalization algorithm*" OR "adaptive intervention*" OR "tailored feedback" OR "fully automated" OR wearable*) OR AB (AI OR "machine learning" OR "deep learning" OR algorithm* OR chatbot* OR "conversational agent*" OR "conversational artificial intelligence" OR "natural language processing" OR "computer-assisted" OR "digital health" OR eHealth OR "mobile health" OR mHealth OR "mobile app*" OR application OR "e-learning" OR "web-based intervention*" OR "text messag*" OR SMS OR "continuous glucose monitoring" OR CGM OR "digital twin" OR "predictive analytics" OR "predictive model*" OR "personalization algorithm*" OR "adaptive intervention*" OR "tailored feedback" OR "fully automated" OR wearable*)) |
| --- | --- | --- |
| 3 | Lifestyle behaviors related to diabetes prevention | (MH "Life Style+" OR MH "Exercise+" OR MH "Diet, Healthy" OR MH "Weight Loss+" OR MH "Nutrition+" OR MH "Obesity+" OR MH "Overweight+" OR MH "Patient Participation+" OR MH "Health Services Accessibility+" OR MH "Rural Health Services+" OR TI (lifestyle OR "physical activity" OR exercise OR diet* OR "weight loss" OR nutrition OR obesity OR overweight OR "abdominal obesity" OR BMI OR "time in range" OR "glucose management indicator" OR "patient engagement" OR "user engagement" OR "program completion" OR "program initiation" OR retention OR reach OR "health professional shortage area*" OR HPSA OR rural OR underserved OR "hard-to-reach") OR AB (lifestyle OR "physical activity" OR exercise OR diet* OR "weight loss" OR nutrition OR obesity OR overweight OR "abdominal obesity" OR BMI OR "time in range" OR "glucose management indicator" OR "patient engagement" OR "user engagement" OR "program completion" OR "program initiation" OR retention OR reach OR "health professional shortage area*" OR HPSA OR rural OR underserved OR "hard-to-reach")) |
| 4 | 1 AND 2 AND 3 | #1 AND #2 AND #3  Limiters:   - English Language - Published Date: 2015–2026 |

493 files imported 8/3/2026

D. Web of Science

| No | Core concept | Search |
| --- | --- | --- |
| 1 | Prediabetes / CKM Stage 1-2 risk | TS=(prediabetes OR "pre-diabetes" OR "prediabetic state" OR "diabetes prevention" OR "type 2 diabetes prevention" OR "impaired glucose" OR "impaired glucose tolerance" OR "impaired fasting glucose" OR IFG OR IGT OR dysglycemia OR "youth-onset type 2 diabetes" OR "adolescents with prediabetes" OR "at risk for type 2 diabetes" OR "elevated diabetes risk") |
| 2 | AI-driven digital health | TI=("artificial intelligence" OR "machine learning" OR "deep learning" OR "AI-driven" OR "AI-powered" OR "AI-based" OR "AI-enabled" OR chatbot* OR "conversational agent*" OR "conversational artificial intelligence" OR "natural language processing" OR "digital health" OR eHealth OR "mobile health" OR mHealth OR "mobile app*" OR "mobile application*" OR "web-based intervention*" OR "text messag*" OR "continuous glucose monitoring" OR CGM OR "digital twin" OR "predictive analytics" OR "predictive algorithm*" OR "personalization algorithm*" OR "adaptive intervention*" OR "tailored feedback" OR "fully automated" OR wearable*) |
| 3 | Lifestyle / CKM Behavioral Targets | TS=(lifestyle OR "physical activity" OR "weight loss" OR "healthy diet" OR "dietary intervention" OR "behavior change" OR "abdominal obesity" OR "time in range" OR "glucose management indicator" OR "patient engagement" OR "user engagement" OR "program completion" OR "program initiation" OR "health professional shortage area*" OR HPSA OR "hard-to-reach") |
| 4 | 1 AND 2 AND 3 | #1 AND #2 AND #3  Limiters:   - Language: English - Publication Years: 2015–2026 |

300 files imported 8/3/2026

E. IEEE Xplore

| 1 | ("prediabetes" OR "pre-diabetes" OR "diabetes prevention" OR "type 2 diabetes prevention" OR "impaired glucose" OR "impaired glucose tolerance" OR "impaired fasting glucose" OR IFG OR IGT OR obesity OR overweight OR "abdominal obesity" OR BMI OR dysglycemia OR "metabolic health" OR "metabolic disease" OR "youth-onset type 2 diabetes" OR "adolescents with prediabetes" OR "at risk for type 2 diabetes" OR "elevated diabetes risk") |
| --- | --- |

| 2 | ("artificial intelligence" OR "machine learning" OR "deep learning" OR "reinforcement learning" OR "large language model" OR LLM OR AI OR algorithm OR chatbot OR "conversational agent" OR "conversational artificial intelligence" OR "predictive model" OR "personalization algorithm" OR "adaptive intervention" OR "mobile app" OR "mobile application" OR smartphone OR "digital platform" OR eHealth OR mHealth OR telehealth OR "web based" OR "online program" OR wearable OR "digital health" OR "text messaging" OR SMS OR "continuous glucose monitoring" OR CGM OR "digital twin" OR "predictive analytics" OR "tailored feedback" OR "fully automated") |
| --- | --- |
| 3 | ("lifestyle" OR "physical activity" OR exercise OR diet OR nutrition OR "weight loss" OR "blood pressure" OR "kidney health" OR eGFR OR "metabolic syndrome" OR "insulin resistance" OR obesity OR overweight OR BMI OR dyslipidemia OR "high cholesterol" OR "time in range" OR "patient engagement" OR "program completion" OR retention OR reach OR "health professional shortage area" OR rural OR underserved) |
| 4 | 1 AND 2 AND 3 |

308 files imported 8/3/2026

F. ClinicalTrials.gov

AREA[ConditionSearch](prediabetes OR "pre-diabetes" OR "prediabetic state" OR "diabetes prevention" OR "type 2 diabetes prevention" OR "impaired glucose" OR "impaired glucose tolerance" OR "impaired fasting glucose" OR IFG OR IGT OR dysglycemia OR "metabolic health" OR "metabolic disease" OR "youth-onset type 2 diabetes" OR "elevated diabetes risk")

AND

AREA[InterventionSearch](AI OR "artificial intelligence" OR "machine learning" OR "deep learning" OR algorithm* OR chatbot* OR "conversational agent" OR "natural language processing" OR "computer-assisted" OR "digital health" OR eHealth OR "mobile health" OR mHealth OR "mobile app" OR application OR "e-learning" OR "web-based intervention" OR "text messaging" OR SMS OR "continuous glucose monitoring" OR CGM OR "digital twin" OR "predictive analytics" OR "predictive model" OR "personalization algorithm" OR "adaptive intervention" OR "tailored feedback" OR "fully automated" OR wearable*)

AND

(AREA[InterventionSearch](lifestyle OR "physical activity" OR exercise OR diet* OR "weight loss" OR nutrition OR obesity OR overweight OR "abdominal obesity" OR BMI OR "patient engagement" OR "user engagement" OR "program completion" OR "program initiation" OR retention OR reach OR HPSA OR rural OR underserved)

OR

AREA[OutcomeSearch](lifestyle OR "physical activity" OR exercise OR diet* OR "weight loss" OR nutrition OR obesity OR overweight OR "abdominal obesity" OR BMI OR "time in range" OR "glucose management indicator" OR "patient engagement" OR retention OR reach))

Searched on 8/3/2026
