# Supplementary material for: AI-Driven Lifestyle Interventions for Reducing Diabetes Risk: Systematic Review
Source: J Med Internet Res. 2026 Oct 15;28:e104940. doi: 10.2196/104940 (PMC13720412; doi:10.2196/104940)
Supplement: Supplementary file 3 [file jmir_v28i1e104940_app3.docx]

## Multimedia Appendix 3. PRISMA-S Checklist (Search Reporting)

| **Item #** | **Topic** | **Checklist item** | **Location / reporting in manuscript** |
| --- | --- | --- | --- |
| 1 | Database name | Name each individual database searched, stating the platform for each. | PubMed, Embase, CINAHL, Web of Science, and IEEE Xplore were each searched individually; platform and dates reported in Appendix 1 and Methods. |
| 2 | Multi-database searching | If databases were searched simultaneously on a single platform, state the platform and list databases searched. | Not applicable; each database was searched individually rather than through a shared multi-database platform. |
| 3 | Study registries | List any study registries searched. | ClinicalTrials.gov was searched for completed, ongoing, or unregistered trials meeting the eligibility criteria. |
| 4 | Online resources and browsing | Describe any online or print source purposefully searched or browsed, and how this was done. | Hand searching of reference lists of related reviews and topic-relevant articles identified during full-text screening yielded 6 additional records. |
| 5 | Citation searching | Indicate whether cited or citing references were examined and describe methods used. | Backward and forward citation searching of included studies was conducted on [date] using [Google Scholar]. |
| 6 | Contacts | Indicate whether additional studies or data were sought by contacting authors, experts, or manufacturers. | Study authors were not contacted for additional data or to identify unpublished studies. |
| 7 | Other methods | Describe any additional information sources or search methods used. | No additional search methods beyond those described in items 1–6 were used. |
| 8 | Full search strategies | Present the full search strategies for all databases and information sources, copied and pasted exactly as run. | Reported in full for all five databases in Appendix 1, including syntax, limits, and hit counts. |
| 9 | Limits and restrictions | Specify limits/restrictions used, or that none were used, and provide justification. | English language and 2015–2025 publication date limits applied across all databases; justification (reflecting increasing adoption of AI applications) reported in Methods. |
| 10 | Search filters | Indicate whether published search filters were used, and cite the filter(s). | No published or validated search filters (e.g., RCT filters) were used. |
| 11 | Prior work | Indicate whether search strategies were adapted or reused from other reviews, citing the source. | The search strategy was developed de novo for this review. |
| 12 | Updates | Report the methods used to update the search(es). | Searches were conducted once, on August 3, 2026, and were not subsequently updated. |
| 13 | Dates of searches | Provide the date when the last search occurred for each source. | August 3, 2026, for all databases; reported in Appendix 1 and Methods. |
| 14 | Peer review | Describe any search peer review process. | The search strategy was peer-reviewed by the review authors. |
| 15 | Total records | Report the number of records identified from each source. | PubMed n=1756; Embase n=5172; CINAHL n=493; Web of Science n=300; IEEE Xplore n=308; hand searching =3; total n=8,032 (Results; Figure 1). |
| 16 | Deduplication | Describe the method(s) used to identify and remove duplicate records. | 1732 duplicate records were removed using Covidence prior to title/abstract screening |

|  |  |  | (Methods; Results). |
| --- | --- | --- | --- |

*Rethlefsen ML, Kirtley S, Waffenschmidt S, et al. PRISMA-S: an extension to the PRISMA Statement for reporting literature searches in systematic reviews. Syst Rev. 2021;10:39.*
