# Supplementary material for: AI-Driven Lifestyle Interventions for Reducing Diabetes Risk: Systematic Review
Source: J Med Internet Res. 2026 Oct 15;28:e104940. doi: 10.2196/104940 (PMC13720412; doi:10.2196/104940)
Supplement: Supplementary file 4 [file jmir_v28i1e104940_app4.docx]

**Multimedia Appendix 4. Data Extraction Form**

# SECTION A: STUDY IDENTIFICATION

1. **First Author:**
2. **Year of Publication:**
3. **Title:**
4. **Journal/Source:**
5. **Country/Region:**
6. **Funding Source:**
7. **Study Type:**
   - Randomized Trial
   - Non-randomized intervention
   - Observational
   - Feasibility/Pilot
   - Protocol
   - Commercial app description
   - Other:

## 8. Setting:

- Community	☐ Primary care	☐ Workplace	☐ Fully remote
- Other:

## 9. Regulatory/Recognition status:

- CDC-recognized
- FDA saMD (Software as a Medical Device)
- Commercial only
- Research Prototype
- Unclear

# SECTION B: POPULATION CHARACTERISTICS

## 10. Target Population:

- Adults with prediabetes
- High-risk individuals
- General population
- Other:
11. **Sample Size:**
12. **Age (mean/range):**
13. **Sex (% female):**
14. **Race/Ethnicity (if reported):**
15. **Equity & Access consideration:**
    - Language options
    - Device requirements
    - Digital literacy
    - Cost barriers
    - Accessibility
    - Others:

# SECTION C: INTERVENTION – AI-DRIVEN APP FEATURES

## C1. Delivery Platform

16. ☐ Mobile app
17. ☐ Web platform
18. ☐ Hybrid (app + web)
19. ☐ SMS-based
20. ☐ Wearable integration
21. ☐ Other:

## C2. Coaching Format

22. ☐ Fully AI/automated
23. ☐ Human coach
24. ☐ Blended AI + human
25. ☐ Chatbot
26. ☐ Peer support

## C3. AI Methodology

27. ☐ Reinforcement Learning
28. ☐ Large Language Model
29. ☐ Rule-based
30. ☐ Hybrid

## C4. Personalization Features

31. ☐ Adaptive goal setting
32. ☐ Tailored feedback
33. ☐ Risk prediction
34. ☐ Recommendation engine
35. ☐ Cultural tailoring

## C5. Engagement Components

36. ☐ Reminders/notifications/push notifications
37. ☐ Gamification
38. ☐ Social support
39. ☐ Progress tracking
40. ☐ Self-monitoring tools

## C6. AI data inputs

41. ☐ Scale
42. ☐ GPS
43. ☐ Wearable
44. ☐ Food log/photo
45. ☐ Electronic medical records
46. ☐ Self-report

## C7. Behavioral Targets

47. ☐ Diet
48. ☐ Physical activity
49. ☐ Weight management
50. ☐ Sleep
51. ☐ Stress
52. ☐ Lipids
53. ☐ Glycemic control
54. ☐ Kidney/eGFR
55. ☐ CVD risk
56. ☐ None

## C8. Cost & Access

57. ☐ Free
58. ☐ Subscription
59. ☐ Insurance covered
60. ☐ Device required

# SECTION D: COMPARATOR

61. ☐ Standard virtual DPP
62. ☐ Usual care
63. ☐ Human coaching
64. ☐ Waitlist
65. ☐ None

# SECTION E: OUTCOMES REPORTED

## E1. Outcome type

66. ☐ Composite
67. ☐ Single clinical
68. ☐ Engagement

## E1. Clinical Outcomes (intervention versus control)

69. ☐ Weight
70. ☐ HbA1c
71. ☐ Blood pressure
72. ☐ Waist circumference

## 73. Results summary:

**E2. Behavioral Outcomes (intervention versus control)**

74. ☐ Diet change
75. ☐ Physical activity
76. ☐ Self-care behaviors

## E3. Engagement Outcomes

77. ☐ Program initiation
78. ☐ Program completion
79. ☐ Engagement

# SECTION G: KEY FINDINGS

80. Main conclusions:
81. Strengths:
82. Limitations:
