# Supplementary material for: AI-Driven Lifestyle Interventions for Reducing Diabetes Risk: Systematic Review
Source: J Med Internet Res. 2026 Oct 15;28:e104940. doi: 10.2196/104940 (PMC13720412; doi:10.2196/104940)
Supplement: Supplementary file 5 [file jmir_v28i1e104940_app5.docx]

## Multimedia Appendix 5. Methodological Quality

| *NHLBI Quality Assessment Tool for Controlled Intervention Studies* |  |  |
| --- | --- | --- |
| Mathioudakis et al (2025) [28] | | Chadwick et al (2025) [25] |
| 1. Was the study described as randomized, a randomized trial, a randomized clinical trial, or an RCT? | Y | N |
| 2. Was the method of randomization adequate (i.e., use of randomly generated assignment)? | Y | NA |
| 3. Was the treatment allocation concealed (so that assignments could not be predicted)? | Y | NA |
| 4. Were study participants and providers blinded to treatment group assignment? | N1 | N1 |
| 5. Were the people assessing the outcomes blinded to the participants' group assignments? | Y | N2 |
| 6. Were the groups similar at baseline on important characteristics that could affect outcomes (e.g., demographics, risk factors, co-morbid conditions)? | Y | N3 |
| 7. Was the overall drop-out rate from the study at endpoint 20% or lower of the number allocated to treatment? | Y | Y |
| 8. Was the differential drop-out rate (between treatment groups) at endpoint 15 percentage points or lower? | Y | Y |
| 9. Was there high adherence to the intervention protocols for each treatment group? | Y | CD^4^ |
| 10. Were other interventions avoided or similar in the groups (e.g., similar background treatments)? | Y | Y |
| 11. Were outcomes assessed using valid and reliable measures, implemented consistently across all study participants? | Y | N5 |
| 12. Did the authors report that the sample size was sufficiently large to be able to detect a difference in the main outcome between groups with at least 80% power? | Y | Y |
| 13. Were outcomes reported or subgroups analyzed prespecified (i.e., identified before analyses were conducted)? | Y | Y |

| 14. Were all randomized participants analyzed in the group to which they were originally assigned, i.e., did they use an intention-to-treat analysis? | Y | N6 |
| --- | --- | --- |
| **Rating** | **Good** | Fair |
| ^1^ NHLBI states that some interventions are difficult to blind^1^ Blinding was not possible due to the nature of the intervention (message content differed by design arm). ^2^The same field workers who delivered the intervention also administered outcome assessments in both arms; assessors were not independently blinded. ^3^ Employment status differed significantly between groups at baseline; other measured characteristics were broadly comparable. ^4^Program-level engagement was reported, but no discrete adherence-to-protocol metric was defined for either arm. ^5^The primary outcome (self-reported physical activity) is subject to recall and social-desirability bias; anthropometric outcomes were measured objectively. ^6^Thirty-four control-group participants who did not complete the WhatsApp opt-in process were  excluded from analysis rather than analyzed as originally assigned. | | |

| *NHLBI Quality Assessment Tool for Observational Cohort and Cross-Sectional Studies* | | | | |
| --- | --- | --- | --- | --- |
|  | Auster-Gussman et al (2022) [29] | Graham et al (2022) [30] | Graham et al (2023) [31] | Veluvali et al (2025) [32] |
| 1. Was the research question or objective in this paper clearly stated? | Y | Y | Y | Y |
| 2. Was the study population clearly specified and defined? | Y | Y | Y | Y |
| 3. Was the participation rate of eligible persons at least 50%? | Y | Y | Y | Y |
| 4. Were all the subjects selected or recruited from the same or similar populations (including the same time period)? Were inclusion and exclusion criteria for being in the study prespecified and applied uniformly to all participants? | N | Y | Y | Y |
| 5. Was a sample size justification, power description, or variance and effect estimates provided? | NR | Y | NR | N |
| 6. For the analyses in this paper, were the exposure(s) of interest measured prior to the outcome(s) being measured? | N | Y | Y | Y |
| 7. Was the timeframe sufficient so that one could reasonably expect to see an association between exposure and outcome if it existed? | Y | Y | Y | Y3 |
| 8. For exposures that can vary in amount or level, did the study examine different levels of the exposure as related to the outcome (e.g., categories of exposure, or exposure measured as continuous variable)? | N/A | Y | Y | Y |
| 9. Were the exposure measures (independent variables) clearly defined, valid, reliable, and implemented consistently across all study  participants? | Y | Y | Y | Y |

| 10. Was the exposure(s) assessed more than once over time? | N | Y | N | Y |
| --- | --- | --- | --- | --- |
| 11. Were the outcome measures (dependent variables) clearly defined, valid, reliable, and implemented consistently across all study participants? | Y4 | Y | Y | Y |
| 12. Were the outcome assessors blinded to the exposure status of participants? | NR | NR | NR | NR |
| 13. Was loss to follow-up after baseline 20% or less? | Y | N6 | Y | Y7 |
| 14. Were key potential confounding variables measured and adjusted statistically for their impact on the relationship between exposure(s) and outcome(s)? | N/A | Y | Y | Y |
| **Rating** | **Fair** | **Good** | **Good** | **Good** |
| ^1^All data were collected using a continuous glucose monitor (CGM) over a 5–14 day period at baseline (AI training phase); therefore, time-in-range (TIR) estimates are based on a relatively short observation window. ^2^Socioeconomic data was based on zip-code. ^3^Over 3,000 participants started the program, only 414 participants were included in the analysis. ^4^Weight change was calculated only among participants who recorded their weight on at least two  occasions. | | | | |

| *NHLBI Quality Assessment Tool for Before-After (Pre-Post) Studies* | | | | | |
| --- | --- | --- | --- | --- | --- |
|  | Everett et al (2018) [33] | Stephens et al (2019) [27] | Sapre et al (2024) [26] | Stein and Brooks (2017) [34] | Zahedani et al (2023) [35] |
| 1. Was the study question or objective clearly stated? | Y | Y | Y | Y | Y |
| 2. Were eligibility/selection criteria for the study population prespecified and clearly described? | Y | Y | Y | Y | Y |
| 3. Were the participants in the study representative of those who would be eligible for the test/service/intervention in the general or clinical population of interest? | Y1 | CD | N11 | N5 | CD^9^ |
| 4. Were all eligible participants that met the prespecified entry criteria enrolled? | N2 | NR | CD12 | N2 | N2 |
| 5. Was the sample size sufficiently large to provide confidence in the findings? | Y | N | N13 | N6 | Y |
| 6. Was the test/service/intervention clearly described and delivered consistently across the study population? | N3 | Y | N14 | Y | Y |
| 7. Were the outcome measures prespecified, clearly defined, valid, reliable, and assessed consistently across all study participants? | Y | Y | N15 | N7 | Y |
| 8. Were the people assessing the outcomes blinded to the participants' exposures/interventions? | NR | NR | NA | NA | NA |
| 9. Was the loss to follow-up after baseline 20% or less? Were those lost to follow-up accounted for in the analysis? | Y | Y | N16 | N8 | N10 |
| 10. Did the statistical methods examine changes in outcome measures from before to after the intervention? Were statistical tests done that provided p values for the pre-to-post changes? | Y | N/A | N17 | Y | Y |
| 11. Were outcome measures of interest taken multiple times before the intervention and multiple times after the intervention (i.e., did they use an interrupted time-series  design)? | N | NXX | N | Y | Y |

| 12. If the intervention was conducted at a group level (e.g., a whole hospital, a community, etc.) did the statistical analysis take into account the use of individual-level data to determine effects at the group level? | N/A | N/A | NA | NA | NA |
| --- | --- | --- | --- | --- | --- |
| **Rating** | **Good** | **Poor** | Poor | Poor | Fair |
| ^1^The study was analyzed as a before-after design, as the requirement for a digital body weight scale (DBWS) for the intervention precluded the inclusion of a control group. ^2^To be considered low bias, the desired answer was no. ^3^Participants were selected based on being more motivated to change their lifestyle. ^4^Participants were exposed to a non-AI text-based system prior to the AI intervention; however, outcomes were not measured before initiation of the AI intervention.^5^Self-selected commercial-app downloaders; representativeness of the broader at-risk population is unclear. ^6^No formal sample-size or power calculation was reported. ^7^Outcome measures (weight, diet) were user self-entered rather than independently verified. ^8^Of 239 initial app users, only 70 (29%) had sufficient data and were classified as active users included in the final analysis, a loss far exceeding the 20% threshold. ^9^Paying users of a commercial program; representativeness of the broader at-risk population is unclear. ^10^Only 1,066 of 2,217 enrolled users (48%) met data-completeness criteria for glycemic analyses, and only 567 (25.6%) for the weight analysis. ^11^Small convenience sample recruited from a single community program; not clearly representative of the broader population of interest. ^12^Consent/enrollment rate among approached participants was not fully reported. ^13^This was an explicitly developmental pilot (n=13) with no sample-size or power justification. ^14^Message delivery fidelity was imperfect; 84% of messages were sent at the intended time. ^15^Reported outcomes are engagement and feasibility metrics rather than validated health or behavior-change outcomes. ^16^Approximately 31% of participants had disengaged from the program by  week 12. ^17^No formal statistical significance testing of change was reported. | | | | | |
