# Supplementary material for: AI-Driven Lifestyle Interventions for Reducing Diabetes Risk: Systematic Review
Source: J Med Internet Res. 2026 Oct 15;28:e104940. doi: 10.2196/104940 (PMC13720412; doi:10.2196/104940)
Supplement: Supplementary file 6 [file jmir_v28i1e104940_app6.docx]

## Multimedia Appendix 6. Weight-Loss and Glycemic Effect Estimates by Study Design

A. RCT (n=1 study — range/median not applicable)

| **Outcome** | **Study** | **Effect estimate** |
| --- | --- | --- |
| Weight (≥4% loss + ≥150  min/week physical activity, threshold) | Mathioudakis et al (2025) [28] | AI-DPP 12.6% vs human-led 12.4%; RD 0.1% (1-  sided 95% CI −5.5%) |
| Weight (≥5% loss, threshold) | Mathioudakis et al (2025) [28] | AI-DPP 16.9% vs human-led 20.0%; RD −3.1%  (1-sided 95% CI −9.7%) |
| Glycemic (≥0.2 percentage point HbA1c reduction, threshold; prediabetes subgroup,  n=130/arm) | Mathioudakis et al (2025) [28] | AI-DPP 26.9% vs human-led 26.9%; RD 0.0% (1-  sided 95% CI −9.1%) |

*Note: these are between-group threshold-based risk differences, not continuous percentage change — not directly comparable to the observational/before-after rows below.*

B. Quasi-Experimental Nonrandomized Comparative Study (n=1 study)

| **Outcome** | **Study** | **Effect estimate** |
| --- | --- | --- |
| BMI change | Chadwick et al (2025) [25] | −0.0 kg/m² (95% CI −0.6 to 0.5; P=.95) |

*Note: Range/median across studies is not computable.*

C. Observational cohort and cross-sectional studies (n=4 studies)

| **Outcome** | **Studies contributing** | **Individual values** | **Range** | **Median** |
| --- | --- | --- | --- | --- |
| Weight, % change | 2 (Graham et al (2022) [30]; Graham et al (2023) [31]) | 4.1%; 4.55–4.96% | 4.1%–4.96% | 4.53% |
| Weight, absolute change (kg) | 2 (Auster-Gussman et al (2022) [29]; Veluvali et al (2025) [32]) | 4.1 kg; 1.5 kg | 1.5–4.1 kg | 2.8 kg |
| Glycemic, TIR change (percentage points) | 1 (Veluvali et al (2025) [32]) | +10.8pp (74.7%→85.5%) | n/a (single study) | n/a |
| Weight/glycemic not reported | None (weight); Auster-Gussman et al (2022) [29]; Graham et al (2022) [30]; Graham et al (2023) [31] (glycemic) | — | — | — |

D. Pre-post studies with or without no control group (n=5

| **Outcome** | **Study** | **Effect estimate** |
| --- | --- | --- |
| Weight, absolute change (kg) | Everett et al (2018) [33] | −1.6 kg (SD 2.5; P<.001) |
| Glycemic, HbA1c change | Everett et al (2018) [33] | −0.1% (IQR −0.2% to 0.1%; P=.04) |
| Glycemic, fasting glucose change | Everett et al (2018) [33] | −1 mg/dL (P=.59, not significant) |
| Weight, % change (Stein and Brooks (2017) [34]; Zahedani et al (2023) [35]) | 2 studies | 2.38%; 2.3% (prediabetes subgroup);  range 2.3%–2.38%; median 2.34% |

| Glycemic, TIR change (percentage points) | Zahedani et al (2023) [35] | +6.2pp among participants with  prediabetes (49.7%→57.4%); single study, range/median not applicable |
| --- | --- | --- |
| Glycemic, hyperglycemic event reduction | Zahedani et al (2023) [35] | 24%–42% reduction (range as originally reported within this single study); range/median not applicable |

*Note: Stephens et al (2019) [27]and Sapre et al (2024) [26]* *reported neither weight nor glycemic outcomes; range/median across studies is not computable for those measures.*
