# Supplementary material for: AI-Driven Lifestyle Interventions for Reducing Diabetes Risk: Systematic Review
Source: J Med Internet Res. 2026 Oct 15;28:e104940. doi: 10.2196/104940 (PMC13720412; doi:10.2196/104940)
Supplement: Supplementary file 7 [file jmir_v28i1e104940_app7.docx]

## Multimedia Appendix 7. SWiM Checklist (Synthesis Without Meta-Analysis)

| **Item #** | **Topic** | **Checklist item** | **Location / reporting in manuscript** |
| --- | --- | --- | --- |
| 1a | Grouping studies for synthesis | Describe, with rationale, the groups used in the synthesis (e.g., population, intervention, outcome, study design). | Studies were grouped by outcome domain (clinical, behavioral, engagement), by AI intervention type, and by population/delivery setting, as described in Data Synthesis. |
| 1b | Changes to groups since protocol | Detail and provide rationale for any changes made to the synthesis groups since the protocol. | Not applicable; no protocol was registered (see Methods, registration). |
| 2 | Standardised metric and transformation methods | Describe the standardised metric for each outcome and any transformation methods used, with rationale. | A single standardised metric was not applied given heterogeneous outcome reporting (e.g., percentage vs. absolute weight change); effect estimates are reported using each study's original metric and summarised descriptively (range/median) by study design in Supplementary Table X. |
| 3 | Synthesis methods | Describe the synthesis method(s) used and provide a rationale for the choice. | A narrative synthesis, organised by outcome domain and AI intervention type, was used given the diversity of study designs and outcome measures precluding meta-analysis. |
| 4 | Criteria to prioritise results | Where applicable, describe criteria used to prioritise particular studies for the main synthesis or conclusions. | Findings from the single RCT were prioritised in interpretation given its stronger internal validity; observational and pre-post studies were treated as descriptive/hypothesis-generating. |
| 5 | Investigation of heterogeneity | Describe methods used to explore variation in effects across studies. | Weight loss and glycemic outcomes are summarised by study design (RCT, observational, before-after) in Supplementary Table X, reporting range and median within each category. |
| 6 | Certainty of evidence | Describe methods used to assess certainty/confidence in the synthesis findings. | Certainty of evidence (e.g., GRADE) was not formally assessed, as this approach is generally applied to pooled quantitative estimates and was judged inappropriate given the narrative synthesis used; methodological quality was instead assessed using the NHLBI tools (Appendix 3). |
| 7 | Data presentation methods | Describe methods used to tabulate or visually display results of individual studies and syntheses. | Results are presented in Tables 2–4 and the PRISMA flow diagram (Figure 1), grouped by outcome domain, AI type, and population/setting. |
| 8 | Reporting results | Describe results in language consistent with the review question, indicating which studies contribute to each synthesis. | Reported by objective in Results (Effectiveness overall; by AI type; by population/setting), citing contributing studies throughout. |
| 9 | Limitations of the synthesis | Report limitations of the synthesis methods and/or groupings used, and how these affect the conclusions. | Beyond the absence of a pooled quantitative synthesis, grouping studies by outcome domain and AI type meant that comparisons across groups were  descriptive rather than statistically tested; |

|  |  |  | observed differences may reflect study design or sample differences rather than true intervention effects (Discussion, Limitations). |
| --- | --- | --- | --- |

*Campbell M, McKenzie JE, Sowden A, et al. Synthesis without meta-analysis (SWiM) in systematic reviews: reporting guideline. BMJ. 2020;368:l6890.*
